# Case report: mutation analysis of primary pulmonary lymphoepithelioma-like carcinoma via whole-exome sequencing

**DOI:** 10.1186/s13000-019-0811-7

**Published:** 2019-06-28

**Authors:** Hong Xuan, Chai Zhengjun, Han Yang, Chen Guohan

**Affiliations:** 1 Department of Thoracic, Shanghai East Hospital, Tongji University, Shanghai, China; 2 Department of Pathology, Shanghai East Hospital, Tongji University, Shanghai, China

**Keywords:** Lymphoepithelioma-like carcinoma, Lung cancer, Whole-exome sequencing, Gene mutation, Tumor mutation burden

## Abstract

**Background:**

Primary pulmonary lymphoepithelioma-like carcinoma (LELC) is a rare tumor subtype accounting for around 0.9% of lung cancers. At present, research on LELC mainly focuses on pathological diagnosis, while the molecular mutation landscape is still unclear.

**Case presentation:**

A 72-year-old female presented a productive cough for three weeks followed by severe symptoms for another week. Respiratory sounds were weak and coarser in the right lung field. F-FDG PET-CTA showed a hypermetabolic mass in the upper lobe of the right lung as well as the enlargement of right hilar and subcarinal lymph nodes. Hematoxylin-eosin staining and immunohistochemistry staining of the biopsy established the diagnosis of primary pulmonary LELC. After thoracoscopic-assisted radical resection of right lung cancer and middle lobe of right lung, the patient’s vital signs were stable without apparent productive cough, chest pain, chest tightness and other subjective discomforts. Furtherwhole exome sequencing of the patient’s tumor tissue and leukocytes (served as a germline mutation control) revealed 613 somatic gene mutations, and of which mutations in *PRIM2, KCNB1, CDH1,* and *ATRX* were most likely related to the LELC pathogenesis. The recurrence of gene mutations from various cancers database and a tumor mutation burden (TMB) of 18.7 mutations/mb were revealed as well.

**Conclusion:**

Our findings have illustrated the genomic profile of a primary pulmonary LELC case and provided a positive biomarker that immune checkpoint blockade is potentially effective for this patient in further treatment.

## Background

Lymphoepithelioma-like carcinoma has been widely described in the nasopharynx, stomach, thymus, liver, cervix, salivary glands and urinary bladder [1]. Primary pulmonary lymphoepithelioma-like carcinoma is a rare subtype which shares histologic features with the undifferentiated (Type III) nasopharyngeal carcinoma (NPC) [2]. Primary pulmonary LELC was first described by Begin et al. in 1987 [3], and approximately 200 cases of LELC of the lung have been reported. It accounts for about 0.9% of lung cancers.

Epstein-Barr virus (EBV) is a lymphotropic virus that belongs to the Herpesviridae family and infects over 90% of adults worldwide. EBV is found to be strongly associated with Asian primary pulmonary LELC patients [4] but not in western patients [5]. Some studies have shown that EBV implied an etiologic role in the pathogenesis of LELCs [4, 6, 7]. Of note, recent researches on LELC mainly focus on pathological diagnosis, while the genomic alterations of pulmonary LELC remain unknown and the treatment strategies are not well-established. Herein, we report a patient with an EBV-associated lung carcinoma showing a LELC pattern and explored its mutation analysis via whole-exome sequencing.

## Case presentation

A 72-year-old Chinese female (non-smoker), who has suffered from blood hypertension for over 30 years, chronic bronchitis for over 20 years, and diabetes for 6 years, was admitted to our hospital due to a productive cough for three weeks followed by severe symptoms for another week.

Respiratory sounds were weak and coarser in the right lung field. Laboratory examination revealed a high percentage of monocytes, a low level of hemoglobinn and a low mean corpuscular hemoglobin concentration. A hypermetabolic mass in the upper lobe of the right lung as well as the enlargement of right hilar and subcarinal lymph nodes were determined by F-FDG PET-CTA, suggesting lung cancer and lymph node metastases. The tumor was measured as 2.8 × 2.2 × 3 cm and ulcerated.

**Fig. 1:**
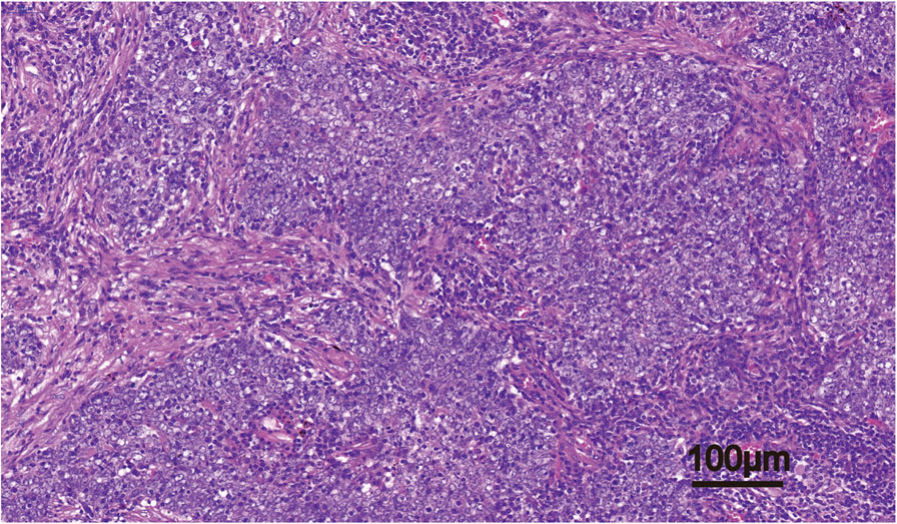
LELC grew infiltrative in the fibrous interstitium and arranged in sheets and syncytial pattern. Neoplastic cells presented vacuolar nucleus with prominent nucleoli and a marked lymphocytic infiltrate. (H&E stain, 200 × magnification)

**Fig. 2:**
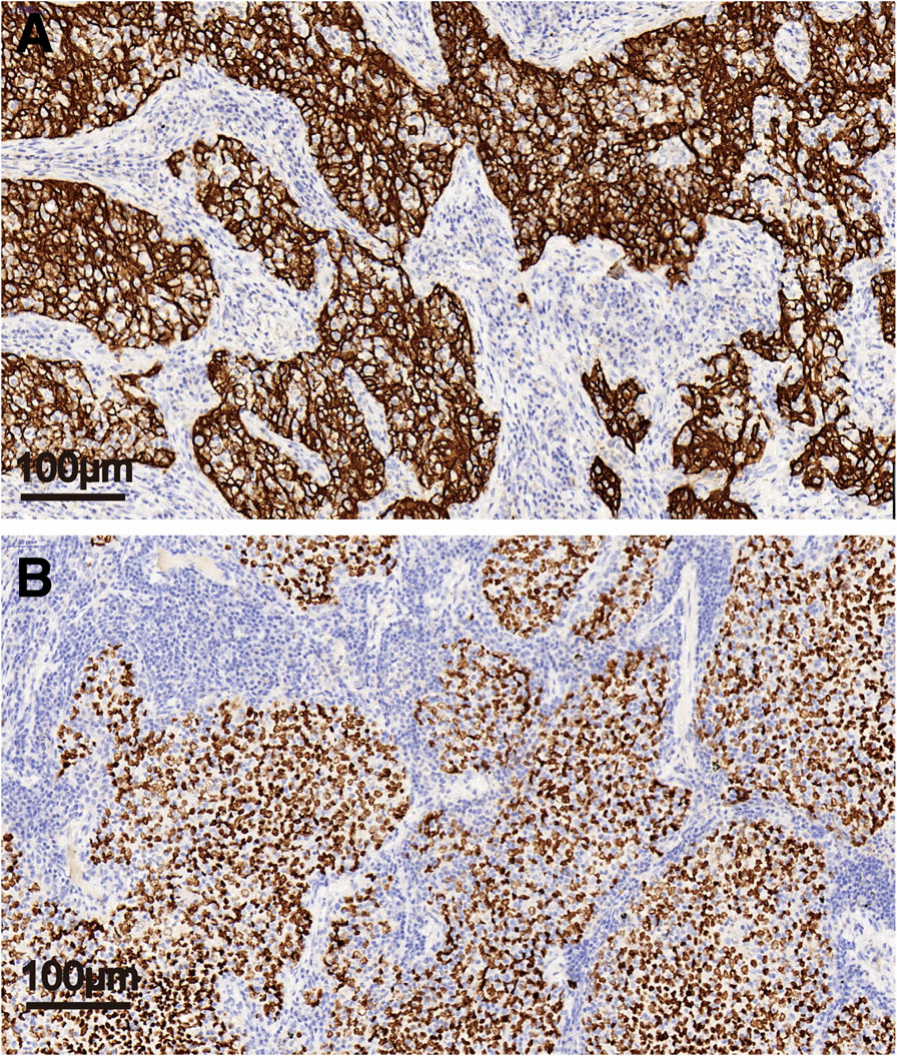
LELC showed strong CK5/6 (**a**) and P40 (**b**) immunoreactivities in neoplastic cells. (IHC stain, 200× magnification)

Routine histologic sections stained with hematoxylin-eosin showed that tumor cells grew infiltrative in fibrous interstitium and arranged in sheets and syncytial pattern with marked pleomorphism. Neoplastic cells presented vacuolar nucleus with prominent nucleoli and a marked lymphocytes infiltration (Fig. 1). Considering the high similarity of histology features between LELC and nasopharyngeal lymphoepithelioma, we first confirmed the absence of a primary lesion in the nasopharynx. Subsequent immunohistochemistry staining was performed on formalin-fixed paraffin sections to confirm the diagnosis. In line with previous LELC reports [8, 9], the tumor cells were strongly positive for CK5/6 and P40 (Fig. 2), excluding the large-cell lymphoma. Besides, the tumor cells showed negative immunostaining of Napsin A, TTF1, CD56, CgA, and Syn, further excluding the possibilities of lung adenocarcinoma and neuroendocrine carcinoma [10, 11].

**Fig. 3:**
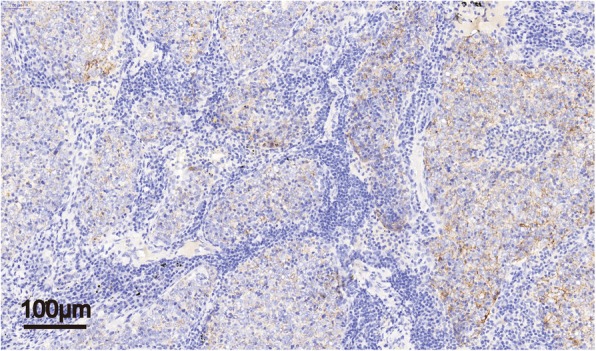
LMP-1 showed granular positivity in LELC neoplastic cells. (IHC stain, 200× magnification)

In addition, latent membrane protein (LMP1) expression of the Epstein-Barr virus was positive in tumor cells (Fig. 3). Chemiluminescence analysis of EB virus antibodies showed that EBV-EA IgA and EBV-VCA IgG were both positive, confirming EBV infection in this patient. The patient was formerly infected by cytomegalovirus (CMV), so the CMV-Ab IgG was positive as well.

Microscopic examination further excluded the vessel invasion, nerve invasion, and pleural invasion. No metastasis was found at the resection margin of the bronchus or in group 7 lymph nodes, but in the bronchial lymph nodes. The patient’s vital signs were stable without apparent productive cough, chest pain, chest tightness and other subjective discomforts after thoracoscopic-assisted radical resection of right lung cancer (right upper lobe lobectomy and lymph node dissection) and resection of middle lobe of right lung.

## Results

The 72-year-old Chinese female patient was eventually diagnosed with primary pulmonary LELC. After obtaining the informed consent from this patient, we further studied the mutation pattern and potential pathogenic gene mutations of tumor tissue via whole-exome sequencing (WES). Fresh-frozen tumor tissue sample was collected for WES, while germline DNA was obtained from peripheral blood mononuclear cells. The raw sequencing data were aligned to the reference human genome (UCSC hg19) through Burrows-Wheeler Aligner [12].

In specific, a total of 611 somatic gene mutations, including 447 nonsynonymous mutations, 125 non-frameshift-deletion, 8 non-frameshift-insertion, 18 frameshift-deletion, 6 frameshift-insertion, 3 stopgain, and 4 stoploss were detected. Among them, mutations in *PRIM2, KCNB1, CDH1,* and *ATRX* were most likely related to LELC pathogenesis (Table 1). No germline mutation associated with pathogenesis was detected. In addition, we also calculated the tumor mutation burden (TMB) as the method previously described [13]. The TMB of this sample is 18.7 mutations/mb.

**Table 1:** Mutations list likely related to LELC pathogenesis

Gene	Position	Transcript ID	Exon	Coding sequence change	Protein change	Variant type
PRIM2	chr6:57398207	NM_000947	10	c.910G > T	p.Gly304*	nonsense
CDH1	chr16:68844233	NM_004360	6	c.821G > A	p.Gly274Asp	missense
ATRX	chrX:76778812	NM_000489	31	c.6767C > G	p.Ser2256Cys	missense
KCNB1	chr20:47991162	NM_004975	2	c.935G > A	p.Arg312His	missense

In order to figure out if there are possible common pathogenic mutations in other cancers, we compared with various cancer mutation data from The Cancer Genome Atlas (TCGA). We found that there were 11 mutations which detected in this LELC samples shared with other cancers (Table 2).

**Table 2:** Consistent mutations shared with the sample from TCGA database

Gene	Position	Transcript ID	Exon	Coding sequence change	Protein change	Variant type	Cancer type
DNMT3A	chr2:25523009	NM_022552	3	c.176delC	p.P59fs	frameshift deletion	STAD, UCEC
PIK3CB	chr3:138413710	NM_001256045	3	c.346delC	p.R116fs	frameshift deletion	UCEC
FGFR3	chr4:1807545	NM_022965	11	c.1378delC	p.P460fs	frameshift deletion	STAD
BRD3	chr9:136918529	NM_007371	2	c.71delC	p.P24fs	frameshift deletion	COAD
BRCA2	chr13:32913428	NM_000059	11	c.4936G > C	p.E1646Q	missense	LUAD
PSMB5	chr14:23502760	NM_001130725	2	c.G13A	p.A5T	missense	PAAD
NOTCH3	chr19:15296358	NM_000435	13	c.2084delC	p.P695fs	frameshift deletion	STAD, UCEC
GNAS	chr20:57415178	NM_016592	1	c.G17A	p.R6Q	missense	HNSC
BCR	chr22:23654017	NM_021574	18	c.G3184A	p.D1062N	missense	STAD

*STAD* Stomach adenocarcinoma

*UCEC* Uterine Corpus Endometrial Carcinoma

*UCS* Uterine Carcinosarcoma

*HNSC* Head and Neck squamous cell carcinoma

*COAD* Colon adenocarcinoma

*LUAD* Lung adenocarcinoma

*PAAD* Pancreatic adenocarcinoma

## Discussion and conclusions

As a rare histology type of lung cancer, primary pulmonary LELC was mainly studied for pathological diagnosis. The majority of primary pulmonary LELCs were usually in early or locally advanced stages and exhibited an improved prognosis and all wild-type EGFR [14]. This case had the similar clinical manifestation and did not habour any EGFR, KRAS and BRAF mutation orALK, ROS1 gene rearrangements, which were were common mutational characteristics for NSCLC, especially for Asian patients. Meanwhile, the molecular mutation landscape has not well developed in primary pulmonary LELC yet. WES analysis provides a potential molecular diagnosis method which could help us find novel gene mutations which may contribute to the malignancy. In this study, we developed the first WES analysis of primary pulmonary LELC and found 611 credible mutations in 340 genes. Notably, mutations in *PRIM2*, *KCNB1*, *CDH1,* and *ATRX* were likely related to the primary pulmonary LELC pathogenesis.

DNA primase has been proved to be essential for the initiation and discontinuous DNA replication. It serves as the polymerase that synthesizes small RNA primers for the Okazaki fragments [15, 16]. PRIM2 encodes a 58 k Da protein which forms the heterodimeric DNA primase enzyme with the p49 subunit. A previous study observed that there were three proviral insertions in PRIM2. Considering the roles of DNA replication initiation for cell proliferation and PRIM2 for DNA replication, proviral activated locus may exert a stimulatory effect on cell growth [16]. A nonsense mutation detected in PRIM2 (c.910G > T) was found in this sample, which might result in the loss of protein function.

As a cancer suppressor gene, CDH1 encodes a transmembrane glycoprotein E-cadherin which maintains Ca^2+^-dependent cell-cell adhesion in epithelial tissues [17, 18]. Altered E-cadherin expression was observed in different types of malignancies, including ovarian cancer [19], colorectal carcinoma [20], prostate cancer [21] and so on. Yoshiura et al. found that the hypermethylation in CDH1 promoter was responsible for E-cadherin inactivation in human carcinomas [22]. Furthermore, CDH1 mutations were identified in approximately 50% of lobular breast cancers and sporadic diffuse gastric tumors [23, 24], indicating the possible roles of CDH1 mutations in E-cadherin inactivation in carcinoma. Exon skippings and the related in-frame deletions are the predominant defects in the diffuse gastric tumor; In contrast, out-of-frame mutations are most mutations found in infiltrating lobular breast cancer [25]. Of note, a missense mutation (c.1679C > G) variant in CDH1 exerted deleterious effects and was associated with hereditary diffuse gastric cancer [26]. These all suggest that an precise genetic counseling is of great value in the diagnosis of CDH1-related carcinomas. A new missense mutation (c.821G > A) variant was found in this patient. The effects of this missense mutation are still unclear and needed to develop functional studies in vitro or in vivo to clarify its potential biological effects.

ATRX (alpha thalassemia/mental retardation syndrome X-linked) gene encodes a transcription regulator protein Atrx which contains a helicase/ATPase domain at the C terminus. As a member of the SNF2 (SWI/SNF) family of chromatin-associated proteins [27], ATRX interacts with death-domain associated protein (DAXX) and contributes to chromatin remodeling at telomeres, where the complex is needed for the deposition of H3.3 at heterochromatin loci [27, 28]. Of note, Heaphy et al. revealed that ATRX mutations were associated with the abnormal telomeres in tumors of the central nervous system [29]. In addition, ATRX mutations occurred in other types of carcinoma, including uterine leiomyosarcoma [30, 31] and pancreatic neuroendocrine tumors [32], and were often correlated with a poor prognosis. An exome sequencing study on uterine leiomyosarcomas (ULMSs) identified ATRX as the second most mutated gene in their study. Of note, all mutations they observed were nonsense mutations, small frameshift insertions and deletions, which all might result in a truncated protein product [30]. By contrast, we found a missense mutation (c.6767C > G) of ATRX in this sample. There are no functional study of this ATRX missense mutation reported, it is of value to explore the function of this detected missense mutations in future studies.

KCNB1 (potassium voltage-gated channel subfamily B member 1) encodes a voltage-gated potassium (K(+)) channel which conducts delayed rectifier current in the brain, pancreas and cardiovascular system [33]. KCNB1 is involved in many important cellular processes, including hippocampal neuron excitation homeostasis, apoptotic programs associated with oxidative stress and the progression of gliomas [33–35]. Numerous studies showed that KCNB1 alternationswere associated with several cancers [36–38]. For instance, decreased expression of KCNB1 was correlated with the increased aggressiveness in primary breast tumors [39]. Of note, Wang et al. revealed that KCNB1 was associated with malignant progression and outcome in gliomas [35]. A missense mutation (c.935G > A) in KCNB1 has been detected in this sample. Since there is no function study of this mutation, whether it is associated with LELC occurring is still needed to be further explored.

There is little exploration of genetic profile of primary pulmonary LELC due to its rarity. Meanwhile, the scarcity of primary pulmonary LELC genomic data to date hinders the understanding of its pathogenesis. Of note, we found 11 mutations in 11 genes detected from the WES could be common pathogenic mutations for various cancers by comparing with the mutations in the TCGA database. However, the exact effects of these mutations are still unclear and whether they are associated with LELC pathogenesis is still required for further study.

LYY et al. [40] reported EBV-positive NPC had a higher percentage of somatic mutation and revealed that majority of NPCs displayed activation of the NF-kB signaling pathway as a result of somatic inactivating mutations in negative regulators of NF-kB. We didn’t found the same feature in this sample, although both of NPC and LELC can be EBV-infection positive. Compared with the gene mutation landscape of NPC, we found this LELC sample only shared a few gene abnormalities (e.g. FGFR3), indicating that they may be occurred through different pathways.

Besides the potential pathogenic gene mutations, this sample also had a relative high TMB value. Studies have shown that high TMB is associated with better responses to the checkpoint blockages in non-small cell lung cancer cohorts [13, 41]. Thus, immune checkpoint inhibitors (such as PD-1/PD-L1) are potentially effective therapy for this patient, which needs to be confirmed in future.

Utilizing the whole-exome sequencing, we presented a primary pulmonary LELC case, with special emphasis on pathogenesis, molecular features and differential diagnosis. Our results illustrated the genomic profile of primary pulmonary LELC and provided a positive biomarker that immune checkpoint blockades were likely to be effective for this patient. Furthermore, it still needs to be improved by expanding the samples sizes to show a comprehensive genomic landscape and molecule pathogenesis of the pulmonary LELC.

## Abbreviations

EBV: Epstein-Barr virus
LELC: lymphoepithelioma-like carcinoma
TCGA: The Cancer Genome Atlas
TMB: tumor mutation burden
WES: whole-exome sequencing
